# Amine-Functionalized Covalent Organic Framework for Efficient SO_2_ Capture with High Reversibility

**DOI:** 10.1038/s41598-017-00738-z

**Published:** 2017-04-03

**Authors:** Gang-Young Lee, Joohyeon Lee, Huyen Thanh Vo, Sangwon Kim, Hyunjoo Lee, Taiho Park

**Affiliations:** 1 Pohang University of Science and Technology (POSTECH), Chemical Engineering, Pohang, 37673 Korea; 2 Korea Institute of Science and Technology, Clean Energy Center, Seoul, 02792 Korea

## Abstract

Removing sulfur dioxide (SO_2_) from exhaust flue gases of fossil fuel power plants is an important issue given the toxicity of SO_2_ and subsequent environmental problems. To address this issue, we successfully developed a new series of imide-linked covalent organic frameworks (COFs) that have high mesoporosity with large surface areas to support gas flowing through channels; furthermore, we incorporated 4-[(dimethylamino)methyl]aniline (DMMA) as the modulator to the imide-linked COF. We observed that the functionalized COFs serving as SO_2_ adsorbents exhibit outstanding molar SO_2_ sorption capacity, i.e., **PI**-**COF**-**m10** record 6.30 mmol SO_2_ g^−1^ (40 wt%). To our knowledge, it is firstly reported COF as SO_2_ sorbent to date. We also observed that the adsorbed SO_2_ is completely desorbed in a short time period with remarkable reversibility. These results suggest that channel-wall functional engineering could be a facile and powerful strategy for developing mesoporous COFs for high-performance reproducible gas storage and separation.

## Introduction

Sulfur dioxide (SO_2_) is emitted from petroleum refineries, power plants burning fossil fuels, sulfide-based metal smelters, and other such industries. Among these, power plants burning fossil fuels have become a major cause of atmospheric pollution, including acid rain and smog^1–4^. Therefore, removing SO_2_ from exhaust flue gases of fossil fuel power plants has attracted increased interest, also because of more stringent environmental regulations. Although a number of technologies for fuel-gas desulfurization (FGD) have been developed using techniques such as lime scrubbing, ammonia scrubbing, and physical absorption via organic solvents, the disadvantages of these processes are prohibitive, including low efficiency and generation of huge amounts of inorganic salts, wastewater, and organic solvents^5–8^. Furthermore, the total concentration of SO_2_ in fuel gases is low (e.g., 0.2 vol% SO_2_), and the physical absorption of SO_2_ under relevant conditions is limited^9^. Therefore, we also deem chemical absorption as being necessary.

In recent years, ionic liquids (ILs), which are composed of cation/anion combinations, have received much attention from researchers owing to their specific properties, including negligible vapor pressure, high thermal and chemical stability, and high loading capacity^10–13^. From these properties, the ILs can easily be functionalized into chemical adsorption processes. In particular, the ILs functionalized by amine groups have exhibited a very high SO_2_ adsorption capacity^14–17^. The chemical interaction between SO_2_ and amine groups in the ILs constructs a charge-transfer complex^18, 19^, which forms relatively unstable ionic structure that can reduce the energy requirement for SO_2_ desorption^20^.

In this respect, guanidinium^21–23^, alkanol amine^24^, 1,4-Diazobicyclo[2,2,2]octane (DABCO)^20^, and azole-based ILs^2, 16, 25, 26^ have been developed as chemical adsorbents for SO_2_. Even though a number of functional groups have been reported in the IL fields, practical applications have not yet been realized, because there has been a huge drawback which is relatively slow SO_2_ absorption rate due to the high viscosity of the ILs^27^. To develop efficient SO_2_ adsorbent processes, channels within which gas can easily pass through^28^ combined with functional amine groups can serve as a strategy for successful and efficient gas adsorbency.

Recently, in the field of gas sorption, covalent organic frameworks (COFs), and metal organic frameworks (MOFs) have attracted interest by researchers due to their high mesoporosity with large surface areas and their straightforward synthetic methods^29–34^. In particular, several gas sorption could be further improved by specific functionalization of COFs via the azide-alkyne click reaction^35, 36^ or the high-throughput ring-opening reaction^37^. Recently, Bein *et al*. have suggested a new functionalization method by introducing modulator agents into the one-pot synthesis^38^.

The storage capabilities of COFs for gases, such as hydrogen^39–41^, methane^39, 42, 43^, ammonia^44^, and carbon dioxide^36, 37, 39^ have also been widely investigated; however, to our knowledge, SO_2_ sorption COFs have yet to be reported and may be one of the most promising areas of SO_2_ sorption research with the potential to dramatically improve performance.

**Figure 1:**
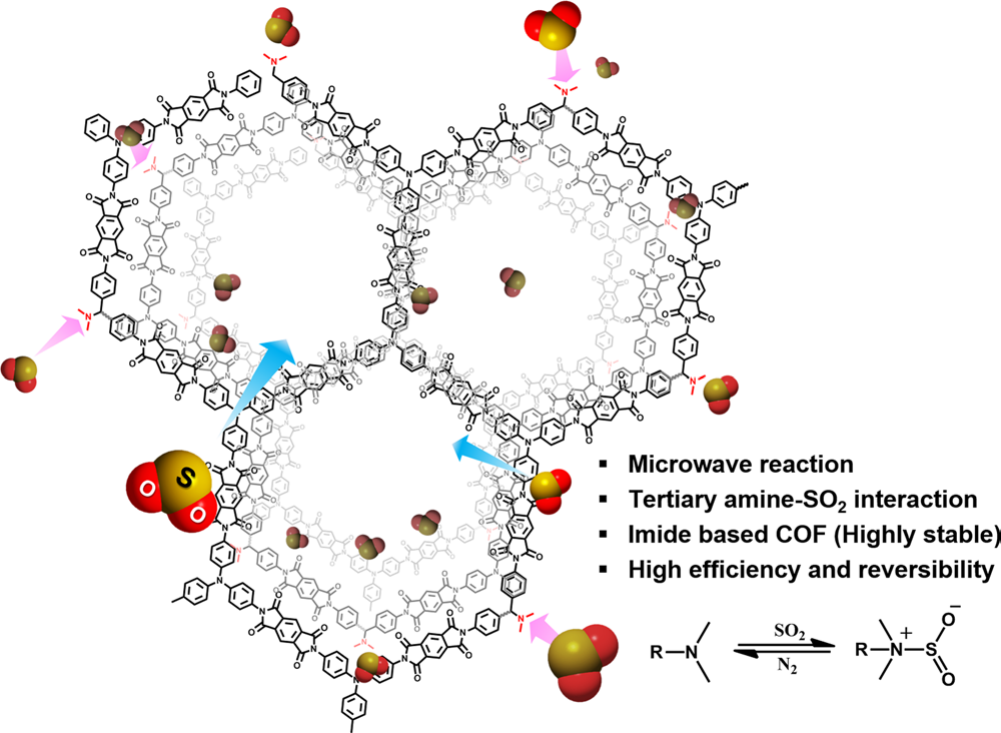
Schematic representation of functionalized PI-COF for SO_2_ sorption.

**Figure 2:**
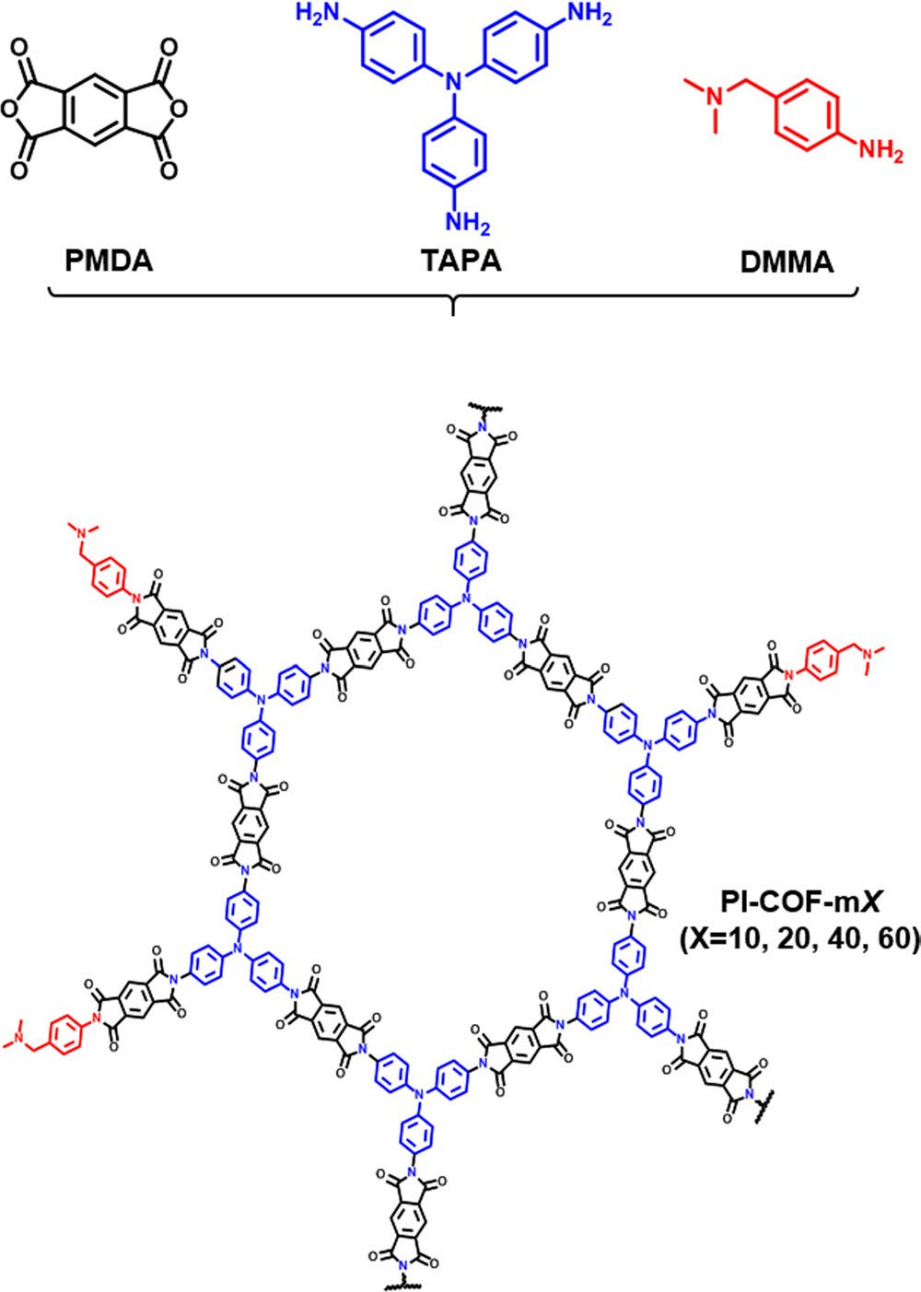
Synthesis scheme of PI-COF-m*X*. PI-COF-m*X* were synthesized via the co-condensation of PMDA (black) and TAPA (blue) with a modulator DMMA (red) serving as an SO_2_ adsorption functional group.

Given the above, we designed a new SO_2_ sorbent using imide-linked COFs (PI-COFs) that have high levels of physical and chemical stability^34^, even though most COFs and MOFs have very weak hydrolytic and thermal stability^45^. Also, quite a lot of SO_2_ sorption MOFs have been reported so far, however, the materials have limitation in long-term stability and reversibility^46–48^. We successfully synthesized a new series of imide-linked COFs that incorporated 4-[(dimethylamino)methyl]aniline (DMMA) as the modulator with various ratios (i.e., *X* = 10, 20, 40, and 60) in Fig. 1, according to a scheme in Fig. 2. The dimethyl functional group of DMMA (i.e., pK_b_ = 4.3) has strong basicity for forming a charge transfer complex with SO_2_ (i.e., pK_a_ = 1.76). Thus, chemical adsorption of SO_2_ could be achieved through the surface-functionalized channel of imide-linked COF.

Furthermore, adsorbed SO_2_ on the imide-linked COF are completely desorbed under desorption conditions, and thus the imide-linked COF can be reused. We were able to obtain functionalized imide-linked COFs via microwave heating in two hours, which was 60 times faster than the five-days reaction time required of the synthesized approach using the conventional solva-thermal method^49, 50^.

## Results and Discussion

### Synthesis of PI-COFs via microwave-assisted reaction

Imide-linked COFs were synthesized as a result of the co-condensation reactions of tris(4-aminophenyl)amine (TAPA) with 1.5 equiv of pyromellitic dianhydride (PMDA)^34^. These two building blocks were then suspended in a 1:1:0.1 mixed solution of N-methyl-2-pyrrolidone (NMP), mesitylene, and isoquinoline under microwave-assisted conditions at 200 °C for 2 h. The advantage of this microwave-assisted reaction is that direct microwave heating is able to reduce chemical reaction times and is also known to reduce side reactions, increase yields, and improve reproducibility of synthesis condition^49–51^.

The condensation reaction of PMDA and TAPA yielded a crystalline brown solid which is insoluble in water and typical organic solvent, such as acetone, hexane, chloroform, tetrahydrofuran (THF), N, N-dimethylformamide, or m-cresol. Among mixed solvents, NMP and mesitylene could control the solubility of building blocks and isoquinoline, being the catalyst, could accelerate reaction time by enhancing the rearrangement of iso-imide to imide^52^. The reversibility of the imidization reactions involves an error-correction mechanism that enables the conversion of kinetic intermediates (amorphous) to thermodynamically stable forms (crystalline)^53^.

FT-IR spectra confirmed C=O groups of the imide rings that corresponded to an asymmetric stretching peak at 1,770 cm^−1^ and a symmetric stretching peak at 1,720 cm^−1^ (see Supplementary Fig. S1). In addition, we observed a stretching vibration of C-N-C groups in the imide at peak 1,375 cm^−1^ and aromatic C-N stretching vibration of the TAPA core at peak 1320 cm^−1^. Furthermore, no bands appeared that corresponded to the starting monomers (i.e., amino around 3,340 cm^−1^ and anhydride at 1,765 cm^−1^) or amic acid intermediate (i.e., amide around 1,650 cm^−1^), demonstrating that the products are fully imidized via the microwave-assisted reaction (**PI**-**COF**-**m**), just as in the solva-thermal method (**PI**-**COF**-**s**). These PI-COFs exhibit high thermal stability regardless of synthetic methods, as determined by thermogravimetric analysis (TGA) (see Supplementary Fig. S2).

### Properties of PI-COFs via microwave-assisted reaction

**Figure 3:**
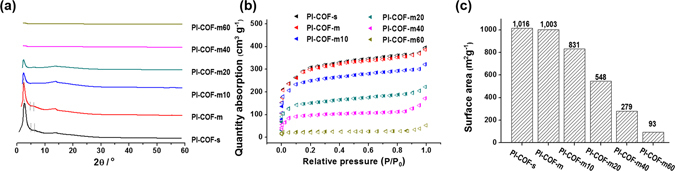
Crystallinity and surface areas of the PI-COF-m*X* series (**a**) a comparison of the synchrotron X-ray scattering profiles of the **PI**-**COF**-**m** ***X*** series; (**b**) nitrogen sorption isotherms of the **PI**-**COF**-**m** ***X*** series recorded at 77 K; and (**c**) BET surface areas obtained from the nitrogen sorption experiments.

In PXRD, the peaks at 3.1°, 5.3°, and 6.2° for both **PI**-**COF**-**s** and **PI**-**COF**-**m** correspond to the (110), (200), and (220) Bragg peaks of the hexagonal network. It is well matched to results presented by Yan *et al*.^34^, as shown in Fig. 3a. The experimental patterns also agreed with simulated PXRD patterns which exhibited eclipsed stacking structure slipped by 1/4 of the unit cell (see Supplementary Fig. S3). This structure has advantage for gas absorption effectively^34^.

We also demonstrated the influence of concentration of building blocks as a driving force for the error correction on the crystalline structure. The best optimized condition was 0.25 M according to the highest crystallinity of **PI**-**COF**-**m** in PXRD (see Supplementary Fig. S4). The appropriate concentration was crucial here, because suitable solubilized building blocks could be polymerized in the crystalline **PI**-**COF**-**m**. Depending on the PXRD data, we concluded that the fully solubilized building blocks would be polymerized in amorphous **PI**-**COF**-**m** because of the insufficient error correction.

Furthermore, we investigated the surface area and pore width of **PI**-**COF**-**s** and **PI**-**COF**-**m** via the Brunauer–Emmett–Teller (BET) method by measuring nitrogen gas (N_2_) sorption at 77 K, revealing reversible isotherms. The discrete step in the sorption isotherms at P/P_0_ = 0.05 could indicate the very well-defined porosity of the frameworks. In addition, the absence of hysteresis during desorption is a common feature of materials containing hexagonally aligned one-dimensional mesopores, which agrees with the crystallinity of frameworks attributed to the PXRD results^54^.

The BET surface area has been recorded up to 1,003 m^2^ g^−1^ with a pore width of 29 Å for **PI**-**COF**-**m**, which is comparable with the 1,016 m^2^ g^−1^ and 29 Å for **PI**-**COF**-**s** ^34^. Scanning electron microscopy (SEM) images of **PI**-**COF**-**s** and **PI**-**COF**-**m** could exhibit homogeneous morphologies, consisting of the aggregation of bid-shaped porous structure (see Supplementary Fig. S5). The above results suggest that our **PI**-**COF**-**m** exhibited hexagonal network, a large surface area, and good morphology, all comparable to the control, **PI**-**COF**-**s**. In addition, the short reaction time of 2 h for **PI**-**COF**-**m** was 60 times faster than the 5 days required by the conventional solva-thermal method.

### Functionalization of PI-COFs with modulators

To functionalize **PI**-**COF**-**m**, we introduced the three equiv of the modulator agents being substituted for fractions *X* of TAPA, as indicated in Fig. 2. In our study, ratio *X* of the modulator was systematically varied from 0% to 60%. The modulator, 4-[(dimethylamino)methyl]aniline (DMMA), which included tertiary amine, that constructs the charge transfer complex with SO_2_, being a relatively unstable ionic structure that could also reduce the energy requirements for SO_2_ desorption, would be the suitable functional building blocks for the SO_2_ sorbent. With the increase in the contents of DMMA in **PI**-**COF**-**m**, the number of functional groups for imidization per the monomers decreased below two, which can interfere with the formation of a network structure and form broken framework (see Supplementary Table S1). With more than 30% of DMMA contents, it is difficult to obtain a porous crystalline structure^55^.

The FT-IR spectra have confirmed chemical functionality attributed to the modulators and pronounced broad peaks at 1,603, 1,450, and 1,250 cm^−1^, which correspond to N-CH_2_ bending, N-CH_3_ bending, and C-N stretching for dimethyl amine group of modulator, respectively (see Supplementary Fig. S6).

### Crystallinity and porosity of functionalized PI-COFs

As shown in Fig. 3a, the crystallinity of **PI**-**COF**-**m** ***X*** has been monitored by PXRD. We observed the successful formation of the hexagonal network of the PI-COFs for up to *X* = 20. As noted above, the number of functional groups for imidization per building block below two, i.e., 1.82 for **PI**-**COF**-**m40** and 1.62 for **PI**-**COF**-**m60**, could not construct the network structures corresponding to the decreased crystallinity. **PI**-**COF**-**m10** and **PI**-**COF**-**m20** revealed broad and relatively decreased peaks at hexagonal Bragg reflection mentioned above, which could be an evidence that the amorphous regions were increased, because the modulator disturbed the construction of the regular stacking of the PI-COFs. Furthermore, we examined the porosity of **PI**-**COF**-**m** ***X*** by measuring nitrogen gas (N_2_) sorption at 77 K, revealing reversible isotherms, as shown in Fig. 3b.

While the modulator-free synthesis produced **PI**-**COF**-**m** with a surface area of 1,003 m^2^ g^−1^, substitution of the modulators decreased the surface area. As shown in Fig. 3c, the surface areas were recorded up to 831 m^2^ g^−1^ for **PI**-**COF**-**m10**, 548 m^2^ g^−1^ for **PI**-**COF**-**m20**, 279 m^2^ g^−1^ for **PI**-**COF**-**m40**, and 93 m^2^ g^−1^ for **PI**-**COF**-**m60**. Because the network could snap due to the outer functional groups and the amorphous regions, which extended and disturbed the formation of well-defined pores, the modulators were increased. In general, PXRD results indicate that the broken network occurs above the 40% ratio. SEM images also indicate that the aggregation of bid-shape porous crystals collapsed as ratio *X* increased for the modulator agents (see Supplementary Fig. S5).

### SO_2_ sorption and desorption on functionalized PI-COFs

**PI**-**COF**-**m** ***X*** was tested as a SO_2_ sorbent to study the effect of functional groups and as a correlation of BET surface and crystallinity with the sorption capacity under anhydrous conditions using an apparatus similar to the one described in literature^27, 56–58^. In a typical experiment, the adsorbent (1 g) was loaded into a 25 mL sorption tube equipped with an electrical heater, temperature controller, and inlet and outlet valves. SO_2_ (99.9%) was introduced into the sorption tube at 25 °C at a rate of 30 mL/min. The weight change during the SO_2_ adsorption was monitored using a balance (accuracy: 0.001) and recorded on a computer until equilibrium was attained. The amount of SO_2_ absorbed by the adsorbent was calculated by subtracting the mass of the initial adsorbent and the mass of SO_2_ in an empty glass tube (0.084 g) from the total mass in the tube. In desorption process, the absorbed SO_2_ was desorbed at 100 °C by flowing N_2_ into the SO_2_ loaded sample at a rate of 30 mL/min. The weight loss during SO_2_ desorption was measured and noted until SO_2_ removed completely.

**Figure 4:**
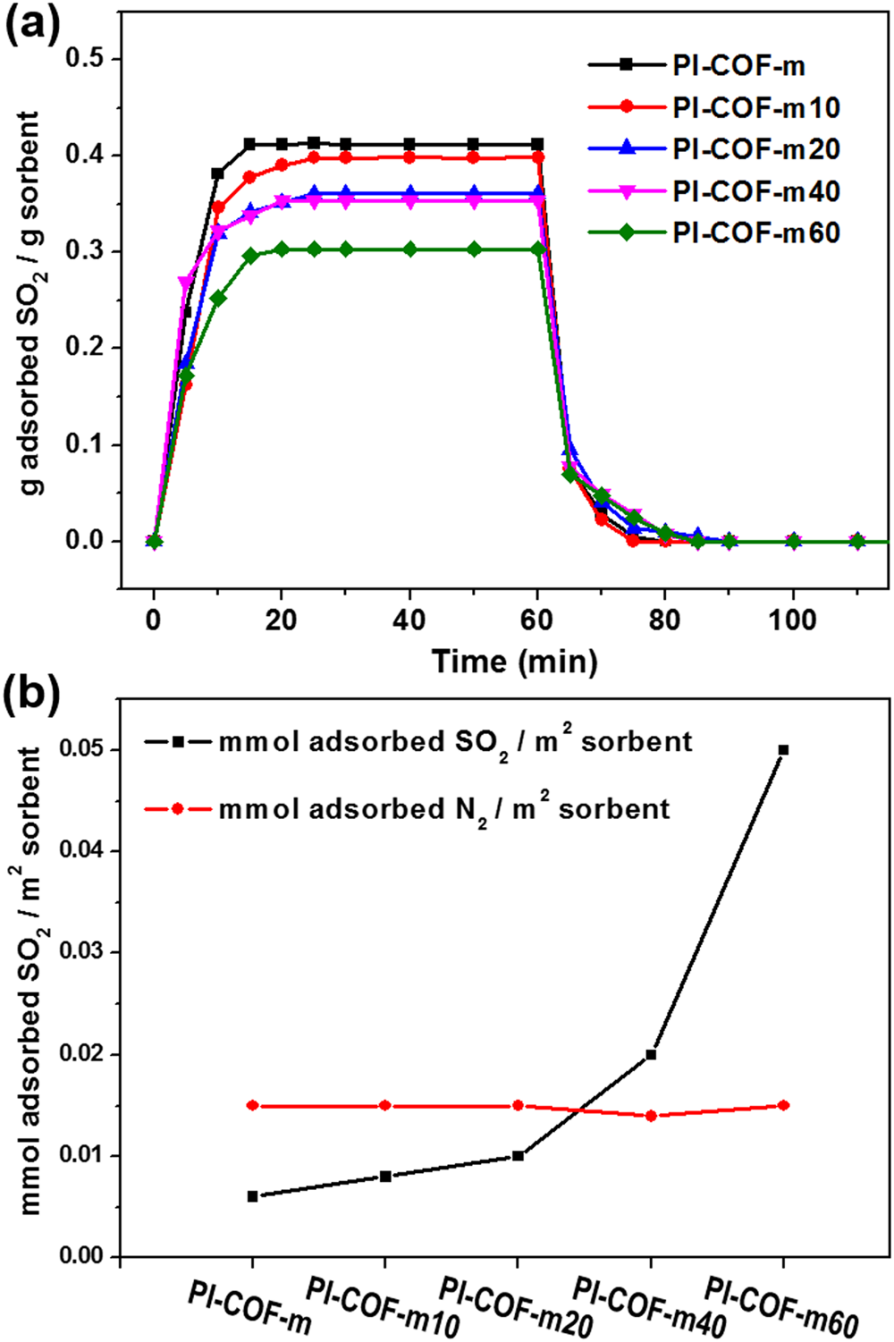
Sorption capacity of PI-COF-m*X* (**a**) A comparison of the SO_2_ sorption and desorption of the **PI**-**COF**-**m** ***X*** series; here, SO_2_ was adsorbed for 60 min at 25 °C at atmospheric pressure, then desorbed at 100 °C for 60 min under flowing N_2_ at a rate of 30 mL/min; (**b**) the effect of surface area on the **PI**-**COF**-**m** ***X*** series for the SO_2_ and N_2_ sorption.

As shown in Fig. 4a, molar SO_2_ sorption capacities of **PI**-**COF**-**m** ***X*** were found to decrease as the ratio of modulators increased. **PI**-**COF**-**m** and **PI**-**COF**-**m10** showed outstanding SO_2_ sorption capacity, recording up to 6.50 mmol SO_2_ g^−1^ (41 wt%) and 6.30 mmol SO_2_ g^−1^ (40 wt%), respectively in Table 1 (see Supplementary Fig. S7). Within a short time period of 20 min, **PI**-**COF**-**m** and **PI**-**COF**-**m10** could adsorb ca. 40 wt% of SO_2_ gas and consistently maintain the sorption capacity just before desorption.

**Table 1:** SO_2_ sorption capacities of the **PI**-**COF**-**m** ***X*** series.

	Capacity^a^ [mmol SO_2_ g^−1^]	Capacity^b^ [g SO_2_ g^−1^]	Capacity^c^ [mmol SO_2_ m^−2^]	Capacity^d^ [mmol N_2_ m^−2^]
PI-COF-m	6.50	0.41	0.006	0.015
PI-COF-m10	6.30	0.40	0.008	0.015
PI-COF-m20	5.64	0.36	0.01	0.015
PI-COF-m40	5.53	0.35	0.02	0.014
PI-COF-m60	4.74	0.30	0.05	0.015

^a^Mmol SO_2_/g sorbent at 25 °C. ^b^G SO_2_/g sorbent at at 25 °C. ^c^Mmol SO_2_/m^2^ sorbent. Unit volume obtained from BET surface in Fig. 3b. ^d^Mmol N_2_/m^2^ sorbent. The molar capacity was calculated based on the BET surface results.

The imide backbone, which is fundamentally polar, provided a great advantage in efficiently absorbing the SO_2_ gas^59^. The large surface area could afford the channels that can be gas was able to pass through. Furthermore, in **PI**-**COF**-**m10**, the strong basic dimethylamine (i.e., pK_b_ = 4.3) could construct charge-complex structures with acidic SO_2_ gas (i.e., pK_a_ = 1.76) attributed to the Lewis base–acid chemical interaction. These structures were relatively unstable forms, i.e., Zwitterions, that could reduce the energy requirement for SO_2_ desorption.

Furthermore, we also have conducted the SO_2_ absorption experiment using water-saturated SO_2_ gas. The concentration of water in SO_2_ flow was 3.5 vol%. The SO_2_ sorption capacity of **PI**-**COF**-**m10** with the water-saturated SO_2_ was 0.35 g SO_2_ g^−1^, that is lower than that of dry SO_2_ (0.4 g SO_2_ g^−1^) (see Supplementary Fig. S8a). However, the capacity was also maintained during 5 cycles. The FT-IR spectrum of the SO_2_ absorbed **PI**-**COF**-**m10** with the water-saturated gas also clearly showed the two peaks centered at 1,323 and 1,145 cm^−1^, corresponding to asymmetric and symmetric SO_2_ stretching peaks, however the intensities of those peaks were lower than those of the one treated with dry SO_2_ (see Supplementary Fig. S8b). This result may indicate that the water block the SO_2_ sorption sites in **PI**-**COF**-**m10**, thereby decreasing the SO_2_ sorption capacity on the sorbent.

### Trade off relation between surface area and functional groups

In addition, **PI**-**COF**-**m20** and **PI**-**COF**-**m40** have almost similar SO_2_ capacity despite the BET surface area of **PI**-**COF**-**m20** being twice as large. This could suggest that there is a trade-off relation between BET surface area and the quantity of functional groups. Even when SO_2_ sorption capacity for **PI**-**COF**-**m60** was recorded at its lowest capacity of 4.74 mmol SO_2_ g^−1^ (30 wt%) among the **PI**-**COF**-**m** ***X*** series, it was still at a high capacity among the reported SO_2_ sorption materials. As shown in Fig. 4b, the SO_2_ molar sorption capacities per the unit surface area of **PI**-**COF**-**m** ***X*** series exponentially increased up to 0.05 mmol SO_2_ m^−2^ for **PI**-**COF**-**m60**, attributing to the decreased BET surface area of 93 m^2^ g^−1^. In contrast, the N_2_ molar absorption capacities per unit surface area of **PI**-**COF**-**m** ***X*** was almost identical regardless of modulator content because there have not been any functional groups for chemical interaction with N_2_. This also indicates that the dimethyl amine group of the modulator is efficient for capturing SO_2_.

**Figure 5:**
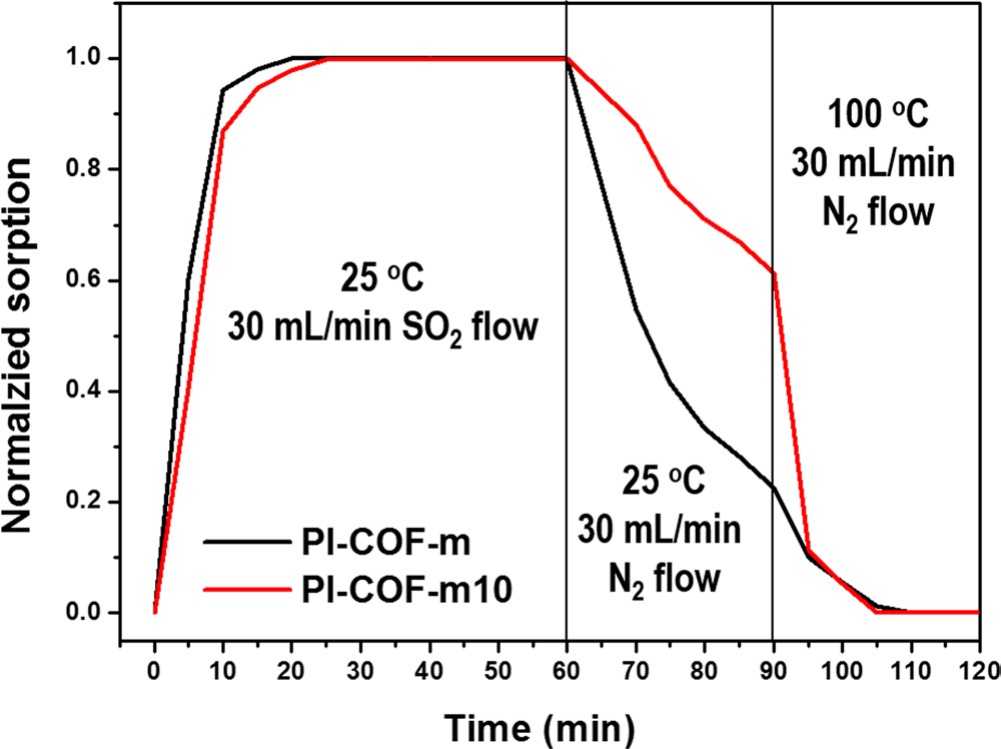
Desorption kinetics of PI-COF-m and PI-COF-m10 versus temperature.

Moreover, as shown in Fig. 5, **PI**-**COF**-**m10** showed much slower desorption kinetics than that of **PI**-**COF**-**m** under 25 °C conditions, resulting from the chemical interaction with SO_2_. Under general desorption conditions at 100 °C, adsorbed SO_2_ was completely desorbed in a short time period.

**Figure 6:**
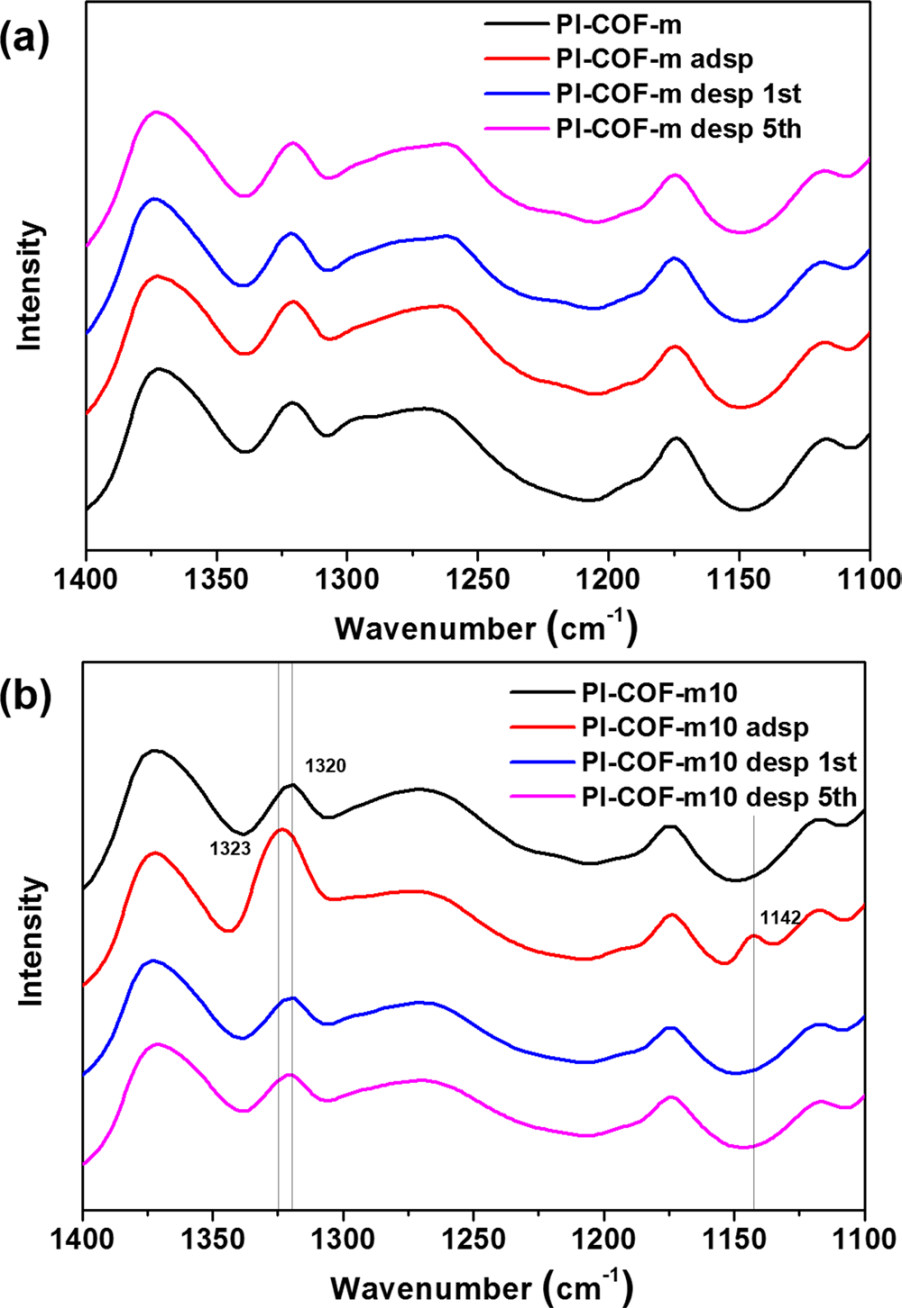
FT-IR spectra of PI-COF-m and PI-COF-m10 after SO_2_ sorption-desorption processing. FT-IR spectra of fresh, SO_2_-loaded, regenerated, and 5^th^ regenerated (**a**) PI-COF-m and (**b**) PI-COF-m10.

Further inspection for the the interaction of **PI**-**COF**-**m** and **PI**-**COF**-**m10** with SO_2_, we investigated FT-IR spectroscopy in Fig. 6. The FT-IR spectrum of **PI**-**COF**-**m** confirmed that there were not any new peaks or shifts upon contact with SO_2_. This suggests that there was no chemical interaction between SO_2_ and **PI**-**COF**-**m**, indicating that **PI**-**COF**-**m** physically absorbed SO_2_ via porous channels. However, for **PI**-**COF**-**m10**, upon contact with SO_2_, we observed new peaks at 1,323 and 1,145 cm^−1^. The appearance of these peaks, being asymmetric and symmetric stretching peaks, respectively, indicates a chemical interaction between SO_2_ and the tertiary nitrogen on the modulators^60^. After the desorption process, these new peaks disappeared, and the peak at 1,320 cm^−1^, attributed to the aromatic C-N stretching vibration of TAPA moiety, reappeared respectively.

### Comparison of the effect of functional groups on SO_2_ sorbent

To clarify the influence of the broken framework resulting from incorporated modulator, we synthesized the PI-COF series with an amine-free modulator, i.e., 4-(tert-butyl)aniline (see Supplementary Fig. S9). These amine-free COFs (**AF**-**COF** ***X***) also exhibit lower crystallinity along with increases of modulator ratio *X*, which is a result of disturbing the construction of the regular PI-COFs (see Supplementary Fig. S10). This trend is consistent with the trend of **PI**-**COF**-**m** ***X***. However, in SO_2_ sorption and desorption tests, we observed that SO_2_ is only physically absorbed on the surface of **AF**-**COF 10** and **AF**-**COF 20** by the FT-IR results (see Supplementary Fig. S11). Thus, we could conclude here that the broken structure from incorporating the amine–free modulator barely influenced on SO_2_ chemical sorption.

### Reversibility of functionalized PI-COF with 10% of modulators

**Figure 7:**
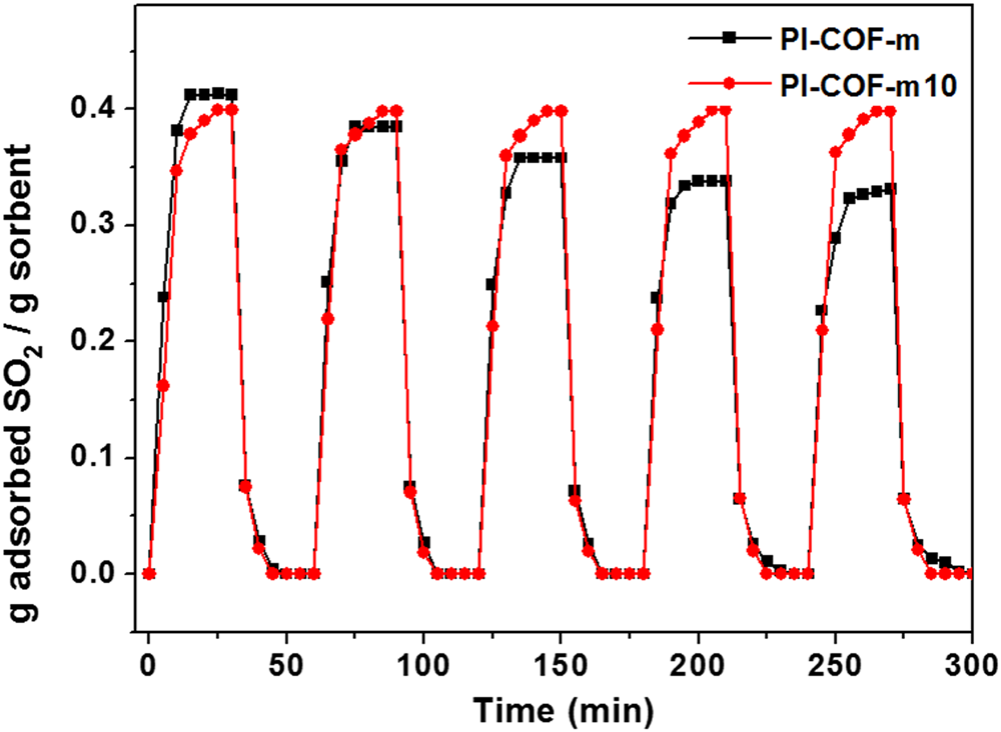
SO_2_ sorption–desorption cycles of the PI-COF-m and PI-COF-m10.

As shown in Fig. 7, we tested the stability and reproducibility of the SO_2_ adsorbency for **PI**-**COF**-**m** and **PI**-**COF**-**m10** through five sorption–desorption cycles. For both sorbents, the release of SO_2_ was completed within 5 min at 100 °C under flowing N_2_. For **PI**-**COF**-**m**, the sorption capacities gradually decreased to 0.33 g SO_2_ g^−1^ (80%) as the cycles repeated. We observed that **PI**-**COF**-**m10** repeatedly recycled without any loss of SO_2_ sorption capacity, indicating that the process of SO_2_ sorption via functionalized **PI**-**COF**-**m10** is completely reversible.

The FT-IR spectrum after five recycled desorption cycles is well matched with the first desorption spectrum, indicating that the adsorbed SO_2_ was entirely desorbed even after several recycle processes. During the recycling test, dramatic change of sample temperature is also noted, which might influence the crystalline structure of **PI**-**COF**s. To demonstrate these crystallinity changes, the PXRD was measured after each of the desorption steps (see Supplementary Fig. S12). The relatively strong crystallinity of **PI**-**COF**-**m** steadily diminished with increasing fast recycle steps in PXRD results; this induced lower SO_2_ sorption capacity than the initial state.

After one day of stabilization in a vacuum, **PI**-**COF**-**m** was recovered its initial crystallinity and SO_2_ capacity, i.e., 0.38 g SO_2_ g^−1^ (93%), by forming the thermodynamically stable crystal structure (see Supplementary Fig. S13). In **PI**-**COF**-**m10**, which has relatively low crystallinity, there was no significant change in crystallinity, thus it was able to retain its initial SO_2_ capacity over several recycling steps (see Supplementary Fig. S12b). This high stability of **PI**-**COF**-**m10** could be attributed to its partially amorphous regions, which is the driving force in effectively maintaining and supporting the framework.

Given the above, a 10%-modulator-substituted **PI**-**COF**-**m10** is the optimal point in the trade-off relation between surface area for physical absorption and amine functional groups for chemical absorption. In other words, a slightly disturbed crystal structure with 10% functionalization in **PI**-**COF**-**m10** could accomplish both outstanding SO_2_ capacity and high reproducibility.

## Conclusion

In conclusion, we successfully synthesized new series of imide-linked COFs that incorporates DMMA as the modulator via a microwave-assisted reaction. The surface-functionalized channel of imide-linked COF for the interaction with SO_2_ was achieved through the dimethyl amine functional groups of DMMA. In addition, well-defined large surface areas could afford the channels that can pass SO_2_ gas through. These imide-based COFs utilizing SO_2_ and tertiary amine-reversible interactions could be perfectly recovered in a recycling process.

Substituted as 10% of the functional group, the molar SO_2_ sorption capacity was recorded up to 6.30 mmol SO_2_ g^−1^ (40 wt%). As the ratio of the modulators increased, molar sorption capacities steadily decreased due to the remarkable decrease in surface area. However, capacities per unit surface area of the **PI**-**COF**-**m** ***X*** series were dramatically increased.

Furthermore, we found that functionalized **PI**-**COF**-**m10** was completely reversible for SO_2_ and highly stable on repeated sorption–desorption cycles. The slightly disturbed crystal structure with 10% tertiary amine functionalization in **PI**-**COF**-**m10** could accomplish both outstanding SO_2_ capacity and the high reproducibility. Our results suggest that channel-wall functional engineering could be a facile and powerful strategy for developing mesoporous COFs for high-performance gas storage and separation.

## Methods

### Materials

All chemicals were purchased from Sigma-Aldrich and used without further purification, except for tetrahydrofuran (THF) and dichloromethane (DCM), which were purified using a J.C. Metyer solvent dispensing system. DMMA was synthesized following the procedures in the Supplementary Information, while **PI**-**COF**-**m** ***X*** was synthesized with the modulator DMMA that we systematically increased from 0% to 60% (see Supplementary Information for details). Finally, SO_2_ (99.9%) and N_2_ (99.9%) were obtained from the Shin Yang Gas Chemical Co.

### Synthesis of PI-COF-s

Pyromellitic dianhydride (PMDA; 165 mg, 0.76 mmol) and tris(4-aminophenyl)amine (TAPA; 140 mg, 0.48 mmol) were evacuated for 2 h and then dissolved in a solution of mesitylene (3 mL)/N-methyl-2-pyrrolidone (NMP) (3 mL)/isoquinoline (0.3 mL) in a glove box under N_2_ atmosphere. The mixed solution was refluxed under a constant flow of nitrogen at 200 °C for five days to afford a brown precipitate, which was isolated by filtration with purified THF (100 mL). The product was immersed in THF (100 mL) for 8 h, during which the activation solvent was decanted and replaced four times. The solvent was removed in a vacuum at 100 °C to afford **PI**-**COF**-**s** as a brown powder (240 mg, 85%). Anal. Calcd for C_66_H_30_O_12_N_8_: C, 84.8; H, 0.32; N, 12.0. Found: C, 83.2; H, 0.49; N, 11.9. FT-IR: 1373, 1505, 1720, 3045 cm^−1^ (see Supplementary Information for further details).

### Synthesis of PI-COF-m

PMDA (165 mg, 0.76 mmol) and TAPA (140 mg, 0.48 mmol) were evacuated for 2 h and then dissolved in a solution of mesitylene (3 mL)/NMP (3 mL)/isoquinoline (0.3 mL) in a glove box under N_2_ atmosphere. The mixed solution was sealed under nitrogen in a 10 mL glass microwave tube, then heated by microwave irradiation at 200 °C with 300 W for 2 h using an Anton Paar microwave synthesizer (monowave 300) to afford a brown precipitate, which was isolated by filtration with purified THF (100 mL). The product was then immersed in THF (100 mL) for 8 h, during which the activation solvent was decanted and replaced four times. The solvent was removed in a vacuum at 100 °C to afford **PI**-**COF**-**m** as a brown powder (227 mg, 80%). Anal. Calcd for C_66_H_30_O_12_N_8_: C, 84.8; H, 0.32; N, 12.0. Found: C, 83.3; H, 0.48; N, 11.6. FT-IR: 1373, 1505, 1720, 3045 cm^−1^ (see Supplementary Information for further details).

### Characterization

^1^H NMR spectra were collected out using a Bruker DPX-300 (300 MHz) FT NMR system operating at 300 MHz. Fourier transform infrared spectra were recorded on a Cary 600 spectrometer equipped with a MCT-A (mercury cadmium telluride) detector with 5 mg samples. Elemental anlyses ware recorded on a Vario ELIII element analysis with 20 mg samples. PXRD data were carried out using a synchrotron radiation on the beam line 5A over the range of 2θ = 1.5–60.0° with a step size of 0.02° and 30 s per step at the Pohang Accelerator Laboratory (PAL), Pohang, Korea. BET data were recorded on a micrometrics ASAP 2010 equipment using N_2_ gas at 77 K. TGA data were obtained under nitrogen atmosphere on a TGA Q50 anlayzer. FE-SEM was performed on a HITACHI S-4800 at 3 keV and 10 μA.

## Electronic supplementary material

Supplementary Information For Amine-Functionalized Covalent Organic Framework for Efficient SO2 Capture with High Reversibility
