# Supplementary material for: Amine-Functionalized Covalent Organic Framework for Efficient SO2 Capture with High Reversibility
Source: Sci Rep. 2017 Apr 3;7:557. doi: 10.1038/s41598-017-00738-z (PMC5429627; doi:10.1038/s41598-017-00738-z)
Supplement: Supplementary file 1 — Supplementary Information For Amine-Functionalized Covalent Organic Framework for Efficient SO2 Capture with High Reversibility [file 41598_2017_738_MOESM1_ESM.pdf]

Supplementary Information

For

Amine-Functionalized Covalent Organic Framework for Efficient SO_2 Capture with High Reversibility

Gang-Young Lee,‡[1] Joohyeon Lee,‡[1] Huyen Thanh Vo,[2] Sangwon Kim,[1] Hyunjoo Lee,*[2] Taiho Park*[1]

[*] Prof. Taiho Park and Dr. Hyunjoo Lee are corresponding-Author

[‡] These authors are contributed equally to this manuscript.

[1] *Chemical Engineering, Pohang University of Science and Technology (POSTECH), 77 Cheongam-Ro, Nam-gu, Pohang, Kyoungbuk, Korea. Fax: +82-54-279-8298; Tel: +82-54-279-2394; E-mail: taihopark@postech.ac.kr*

[2] *Clean Energy Center, Korea Institute of Science and Technology, 5 Hwarang-ro 14-gil, Deongbuk-gu, Seoul 02792, Korea*

Contents

Abbreviations

Experimental details

Figure and Table legends

Table S1. Applied initial weights for the synthesis of **PI-COF-m*X*** with DMMA and average molar functionality of **PI-COF-m*X*.**

Figure S1. FT-IR spectra of PMDA, TAPA, **PI-COF-s,** and **PI-COF-m**.

Figure S2. TGA data of PI-COF series.

Figure S3. Experimental and calculated PXRD patterns of **PI-COF-s** and **PI-COF-m**.

Figure S4. PXRD diffraction patterns of **PI-COF-m** with different concentrations.

Figure S5. SEM images of **PI-COF** series.

Figure S6. FT-IR spectra of **PI-COF-mX**.

Figure S7. A comparison of the SO_2 adsorption and desorption of the **PI-COF-mX** series (mmol adsorbent SO_2 / g adsorbent).

Figure S8. SO_2 sorption and reversibility of **PI-COF-m10** under water saturated condition.

Figure S9. Synthesis of **AF-COF X**

Figure S10. (a) FT-IR spectrum (b) PXRD of **AF-COF 10** and **AF-COF 20.** (C) Comparision of desorption kinetics of **PI-COF-m10**, **AF-COF 10**, **AF-COF 20**.

Figure S11. FT-IR spectra of fresh, SO_2-loaded, regenerated, and compared with adsorbed SO_2 **PI-COF-m10** of (a) **AF-COF 10** (b) **AF-COF 20**.

Figure S12. Comparison of the crystallinity of (a) **PI-COF-**m (b) **PI-COF-m10** as repeating the absorption/desorption cycles.

Figure S13. SO_2 adsorption-desorption cycles of **PI-COF-m** and **PI-COF-m10** after stabilization.

ABBREVIATIONS

COF = covalent organic frameworks; PMDA = pyromellitic dianhydride; TAPA = tris(4-aminophenyl)amine; DMMA = 4-[(dimethylamino)methyl]aniline; FT-IR = Fourier transform infrared spectroscopy; PXRD = powder X-ray diffraction; BET = Brunauer, Emmett, Teller; HRTEM = high resolution transmission electron microscopy; TGA = thermogravimetric analysis; FE-SEM = field emission scanning electron microscopy;

Experimaental details

Materials

All chemicals were purchased from Sigma-Aldrich and used without further purification except tetrahydrofuran (THF) and dichloromethane (DCM) which were purified using J.C. Metyer solvent dispensing system.

Synthesis

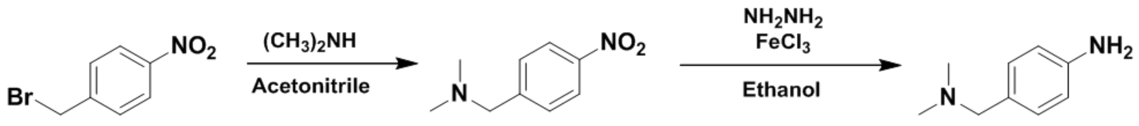

a b c

Synthesis of N,N-dimethyl-1-(4-nitrophenyl)methanamine (b)

To a stirred solution of 1-(bromomethyl)-4-nitrobenzene (compound a; 5 g, 23.1 mmol) in acetonitrile (30mL) was added exessive compound dimethylamine (25 mL). The mixture was stirred at room temperature for 3 h, water (50 mL) was added, and extracted two times with DCM. The organic phased was dried over sodium sulfate, filtered, and concentrated to obtain yellow oil compound b (3.5 g, 83 %). ^{1}H NMR (300MHz, $CDCl_3$) δ 8.20-8.14 (m, 2H), 7.52 (d, *J*=8.7Hz, 2H), 3.38 (s, 2H), 2.28 (s, 6H)

Synthesis of 4-((dimethylamino)methyl)aniline (DMMA, c)

A mixture of compound a (3.5 g, 19.4 mmol) in ethanol was heated to 65 °C. $FeCl_3{\bullet}6H_2O$ (0.83 g, 0.39 mmol) and activated carbon (0.07 g, 5.85 mmol) were added, and 80% hydrazine hydrate (9.8 g, 194 mmol) was added drop wise at such a rate to keep the temperature below 70 °C. The reaction was geated at reflux for 5h and then cooled to room temperature and concentrated. Water (50 mL) was added, the reaction solution was extracted three times with DCM. The organic extracts were combined, dried over sodium sulfate, filtered, and concentrated to obtain colorless oil compound c (3.2 g, 75%). ^{1}H NMR (300 MHz, $CDCl_3$) δ 7.10 (d, *J*=8.3Hz, 2H), 6.30 (d, *J*=8.4Hz, 2H), 3.60 (s, 2H), 3.30 (s, 2H), 2.21 (s, 6H)

Synthesis of PI-COF-mX

A 10 mL microwave glass tube was filled with PMDA, TAPA, and DMMA according to Table S1. The reagent were evacuated for 2 h and dissolved in a solution of mesitylene (3 mL)/NMP (3 mL)/isoquinoline (0.3 mL) in a glove box under N_2 atmosphere. The mixed solution was sealed under nitrogen in the glass microwave tube and heated by microwave irradiation at 200 ^{o}C for 2 h with 300 W using an Anton Paar micro-wave synthesizer (monowave 300) to afford a brown precipitate, which was isolated by filtration with purified THF (100 mL). The product was immersed in THF (100 mL) for 8h, during which the activation solvent was decanted and replaced four times. The solvent was removed under vacuum at 100 ^{o}C to afford **PI-COF-m*X*** as a brown powder. For **PI-COF-m10** Anal. Calcd for $C_{67.8}H_{34.8}O_{12.0}N_{8.40}$: C, 84.2; H, 0.36; N, 12.2. Found: C, 83.6; H, 0.51; N, 12.2. For **PI-COF-m20** Anal. Calcd for $C_{69.6}H_{39.6}O_{12.0}N_{8.80}$: C, 83.7; H, 0.40; N, 12.3. Found: C, 82.8; H, 0.52; N, 12.3. For **PI-COF-m40** Anal. Calcd for $C_{73.2}H_{49.2}O_{12.0}N_{9.60}$: C, 82.7; H, 0.47; N,

12.6. Found: C, 82.2; H, 0.57; N, 12.4. For **PI-COF-m60** Anal. Calcd for $C_{76.8}H_{58.8}O_{12.0}N_{10.4}$: C, 81.8; H, 0.53; N, 12.9. Found: C, 80.9; H, 0.60; N, 12.8.

	Molar ratios (PMDA:TAPA:DMMA)	PMDA (mg)	TAPA (mg)	DMMA (mg)	Quantity (mg)	Average molar functionality
PI-COF-m10	3 : 1.8 : 0.6	165	132	23	230	2.22
PI-COF-m20	3 : 1.6 : 1.2	165	117	46	220	2.06
PI-COF-m30	3 : 1.4 : 1.8	165	103	70	220	1.93
PI-COF-m40	3 : 1.2 : 2.4	165	88	90	200	1.82
PI-COF-m50	3 : 1.0 : 3.0	165	73	114	180	1.71
PI-COF-m60	3 : 0.8 : 3.6	165	60	137	150	1.62
PI-COF-m70	3 : 0.6 : 4.2	165	44	160	80	1.53

Table S1. Applied initial weights for the synthesis of **PI-COF-m*X*** with DMMA and average molar functionality of **PI-COF-m*X***

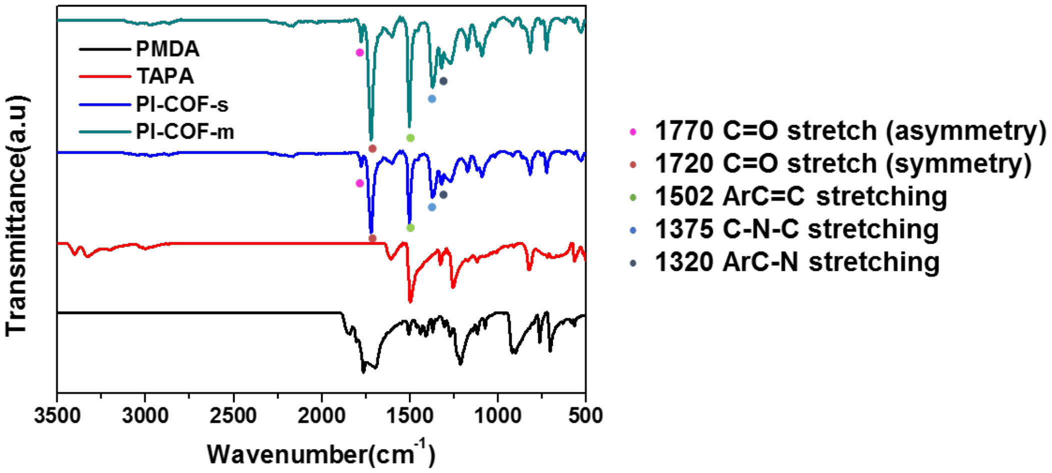

Figure S1. FT-IR spectra of PMDA, TAPA, **PI-COF-s**, and **PI-COF-m.**

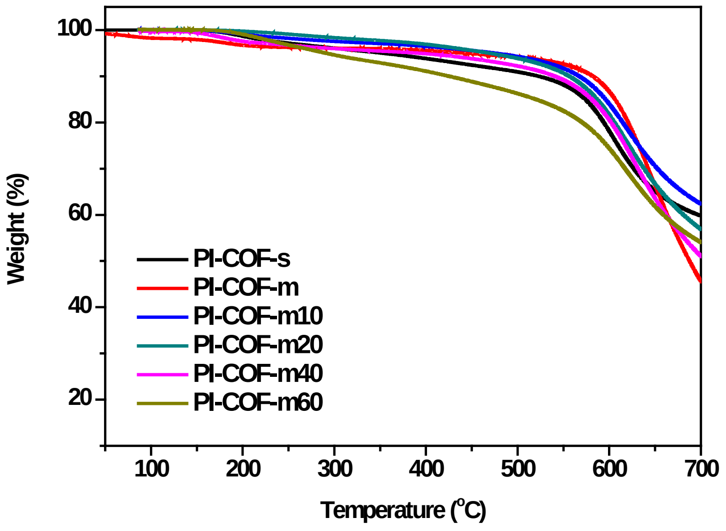

Figure S2. TGA data of **PI-COF** series.

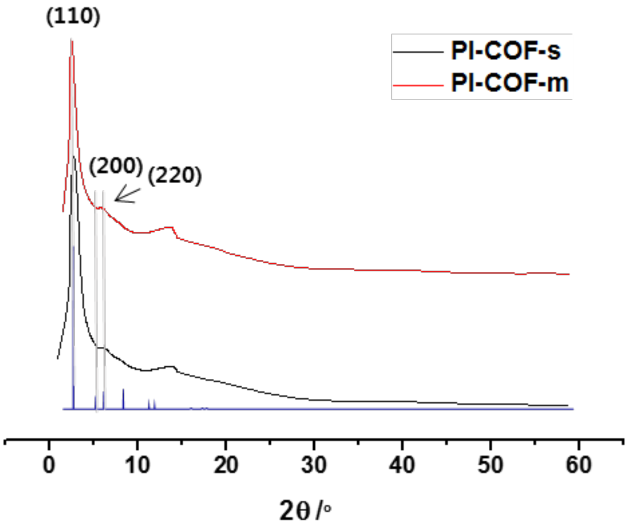

Figure S3. PXRD patterns of **PI-COF-s** with observed in black and **PI-COF-m** with observed in red, calculated based on the serrated stacking bnn net in blue.

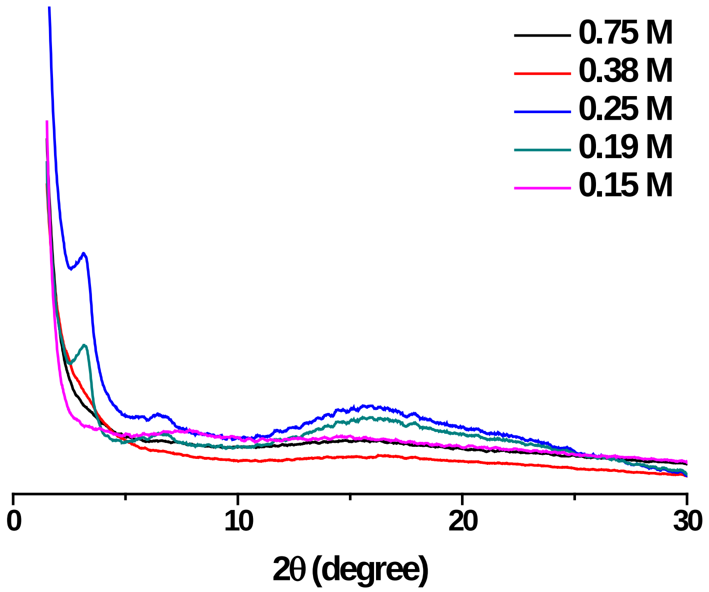

Figure S4. PXRD diffraction patterns of **PI-COF-m** with different concentrations.

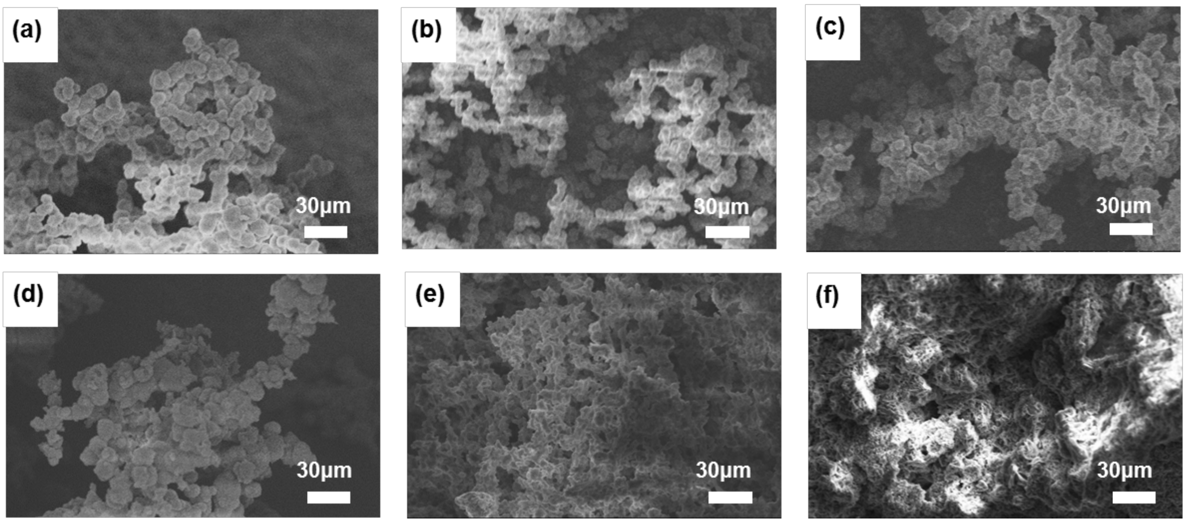

Figure S5. SEM images. (a**) PI-COF-s** (b) **PI-COF-m** (c) **PI-COF-m10** (d) **PI-COF-m20** (c) **PI-COF-m40** (d) **PI-COF-m60**

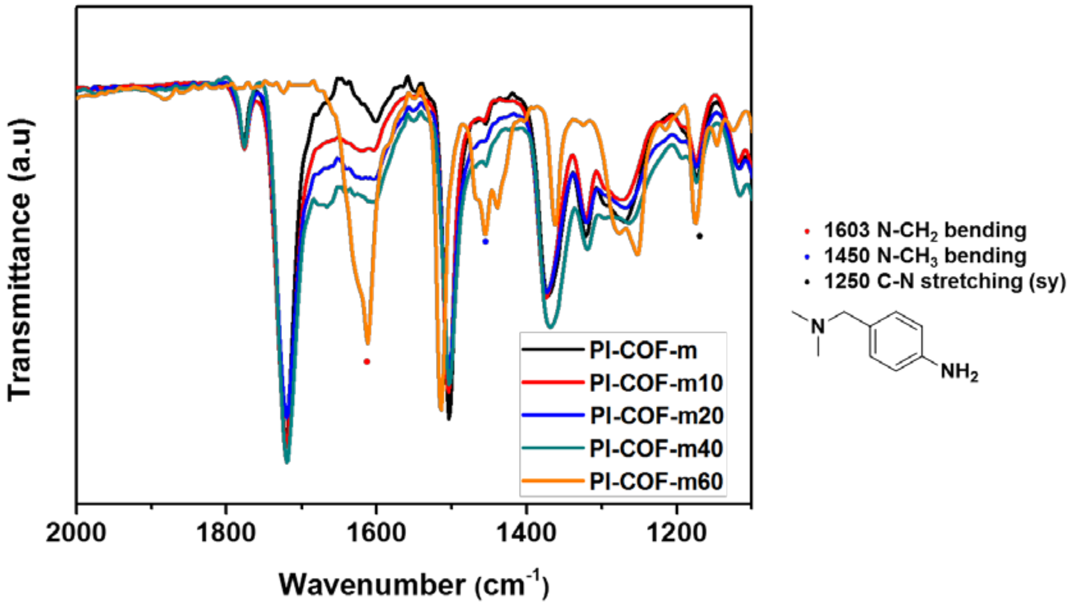

Figure S6. FT-IR spectra of **PI-COF-m*X*.**

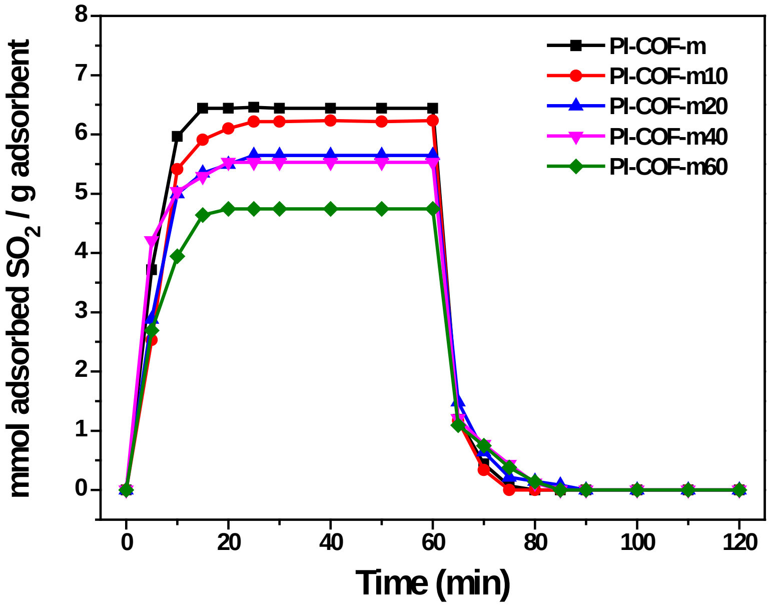

Figure S7. A comparison of the SO_2 adsorption and desorption of **PI-COF-m*X*** series (mmol adsorbed SO_2 / g adsorbent) ; here, SO_2 was adsorbed for 60 min at 25 °C at atmospheric pressure, then desorbed at 100 °C for 60 min under flowing N_2 at a rate of 30 mL/min.

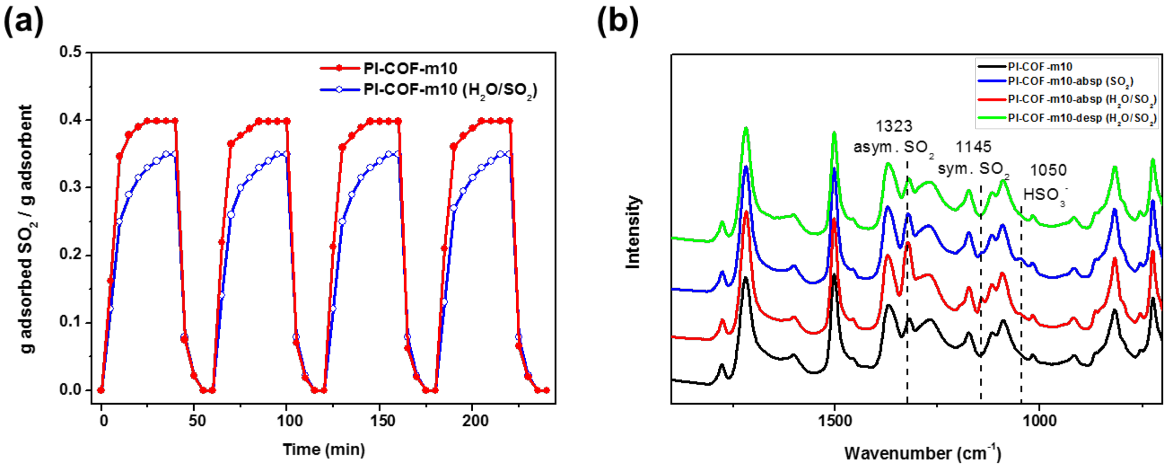

Figure S8. (a) The SO_2 adsorption–desorption cycles of the **PI-COF-m10** with dry SO_2 (red) and water-saturated SO_2 (blue). (b) FT-IR spectra of fresh, dry SO_2, water-saturated SO_2 loaded and regenerated **PI-COF-m10** in water-saturated condition.

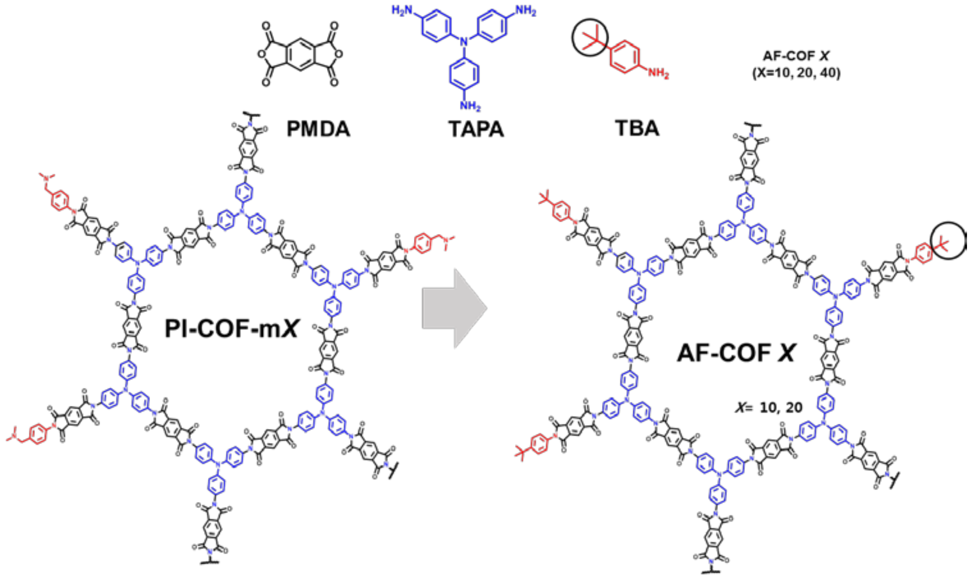

Figure S9. Synthesis of **AF-COF *X*** via the co-condensation of PMDA (black) and TAPA (blue) with a modulator 4-(tert-butyl)aniline (red).

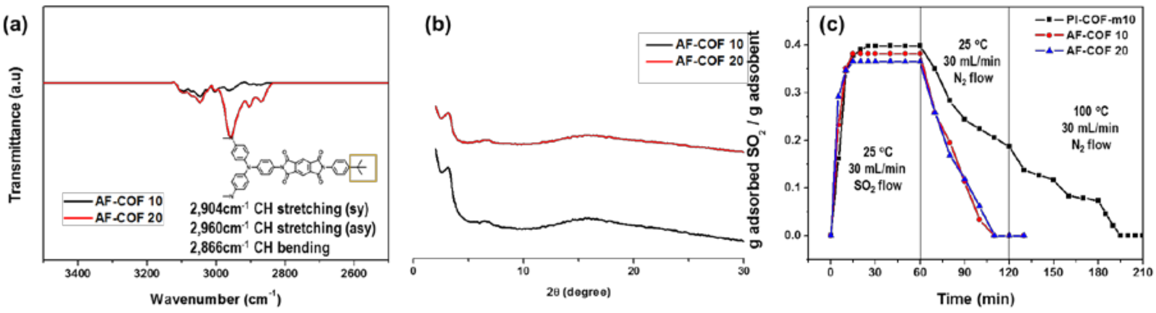

Figure S10. (a) FT-IR specra (b) PXRD of **AF-COF 10** and **AF-COF 20**. (C) A comparison of desorption kinetics of **PI-COF-m10**, **AF-COF 10**, **AF-COF 20**.

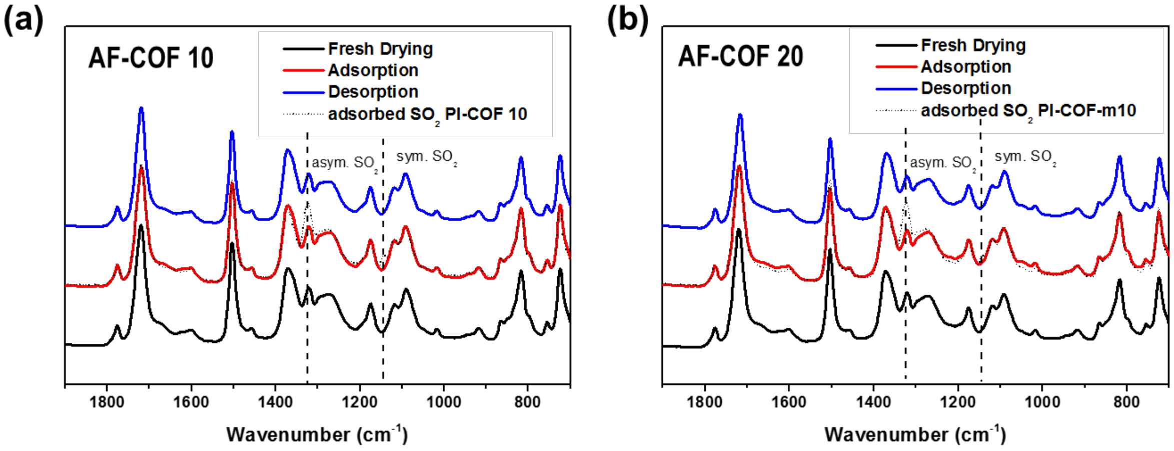

Figure S11. FT-IR spectra of fresh, SO_2-loaded, regenerated, and compared with adsorbed SO_2 **PI-COF-m10** of (a) **AF-COF 10** (b) **AF-COF 20**. There weren't any new peaks or shift upon contact with SO_2. It suggested that there is no chemical interaction between SO_2 and **AF-COF *X***.

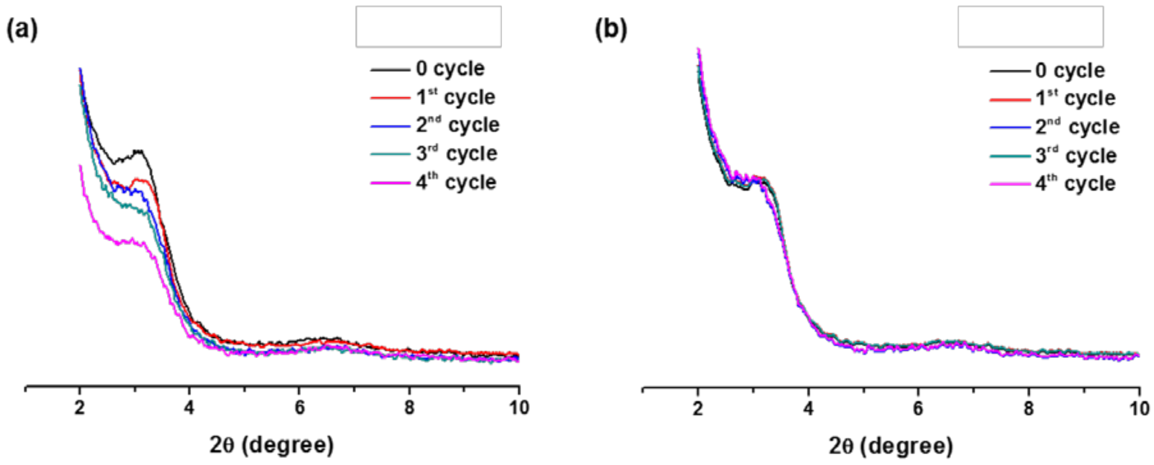

Figure S12. A comparison of the crystallinity of (a) **PI-COF-m (b) PI-COF-m10** as repeating the absorption/desorption cycles.

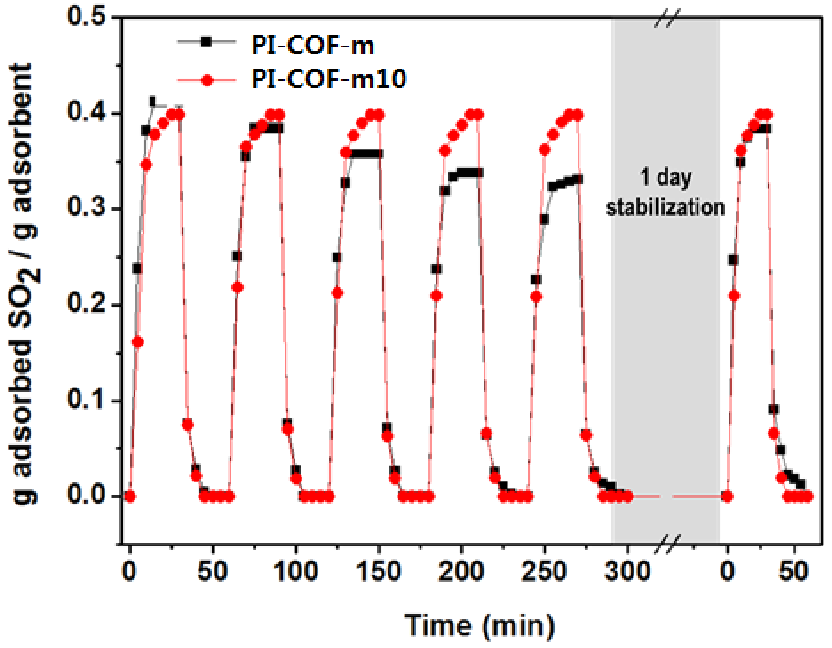

Figure S13. SO_2 adsorption-desorption cycles of **PI-COF-m** and **PI-COF-m10**. The SO_2 adsorption capacity of **PI-COF-m** was recovered to 0.38 g SO_2 g^{-1} (93%) after one-day stabilization.
